# Quantification of Phenolic and Flavonoid Content, Antioxidant Activity, and Proximate Composition of Some Legume Seeds Grown in Nepal

**DOI:** 10.1155/2022/4629290

**Published:** 2022-08-29

**Authors:** Khaga Raj Sharma, Govinda Giri

**Affiliations:** Central Department of Chemistry, Tribhuvan University, Kirtipur, Kathmandu, Nepal

## Abstract

This study was carried out to evaluate some legume seeds growing in Nepal for their proximate composition, quantification of total phenolic (TPC) and flavonoid (TFC) contents, and *in vitro*, antioxidant and antidiabetic activities. These included legume grains such as chickpeas (*Cicer arietinum*), pea (*Pisum sativum*), mung bean (*Vigna mungo*), lima bean (*Phaseolus lunatus*), broad bean (*Vicia faba*), lentil (*Lens culinaris*), soybean (*Glycine max*), and common bean (*Phaseolus vulgaris*). The legume seeds were ground to make the flour which was extracted with methanol. The phenolic and flavonoid content was estimated by Folin-Ciocalteu's phenol and aluminum chloride colorimetric methods. The *in vitro* antioxidant and antidiabetic activity was evaluated by using DPPH (1,1-diphenyl-2-picrylhydrazyl) free radical scavenging and *α*-amylase enzyme inhibition assay. The different legumes showed considerable variations in their phenolic contents (30.64 ± 1.50 mg·GAE/g to 46.65 ± 1.25 mg·GAE/g legume seeds). Similarly, the total flavonoid contents showed 135.5 ± 10.88 mg·QE/g to 191.7 ± 8.73 mg·QE/g legume seeds. The *in vitro* antioxidant activity was evaluated in IC_50_ which ranged from 31.60 ± 0.06 *μ*g/mL to 69.74 ± 0.89 *μ*g/mL. The *α*-amylase inhibition was evaluated in IC_50_ which ranged from 217.38 *μ*g/mL to 425.75 *μ*g/mL as compared to the standard acarbose of 52.76 *μ*g/mL. This study suggested that legumes are good sources of proteins, carbohydrates, and fats mainly for vegetarian people. The selection of the right legume species could be a good source of natural antioxidants and antidiabetics for nutraceutical uses and the beneficial effects of legumes from human health perspectives. Legume seeds growing in Nepal could be used as a sustainable and cheap meat alternative and are considered the most important food source.

## 1. Introduction

Nepal is rich in biodiversity which permits the production of different crops, especially grain legumes. In recent years, legumes have gained high dietary importance due to their known health benefits. Grain legumes are good sources of proteins, carbohydrates, and fats mainly for vegetarian people. Moreover, all farmers in Nepal grow one or more species of pulses or grain legumes. Due to its ecological diversity, Nepal has a great range of productivity of different cereals and pulses. Legumes are the source of biologically active secondary metabolites such as phenols, flavonoids, tannins, alkaloids, saponins, trypsin, glycosides, and coumarins as health-promoting food nutrients [1]. These secondary metabolites show numerous activities in humans and animals as anticancer, antidiabetes, reducing risk of cardiovascular disease, antioxidant, etc. Legumes are known as the poor man's meat, and beans are considered a staple food for vegetarians which most health organizations recommend for frequent consumption of legumes [1].

In recent years, legumes have gained high dietary importance due to their known health benefits. Legume seeds have significantly higher protein content than cereal grains. Hence, legumes are the richest food source of proteins and amino acids for human nutrition [2]. The starches found in legumes are rich in slowly digestible-resistant starch (RS) having a low glycemic index and acting as functional foods [3]. Legumes are rich in dietary fibers content and lower carbohydrates which offers to improve the nutritional quality [4]. The chemical compounds in different legumes differ from species to species with a variety of geographical regions. Legumes are rich in natural bioactive substances such as lectins, enzyme inhibitors, oxalates, oligosaccharides, phytic acids, and phenolic compounds [5]. Legume grains are rich in protein, starch, and fiber ingredients which have a diverse applications such as in packaging and drug delivery, nutraceutical application, 100% gluten-free products, flour mixed doughs and baked foods, meat alternatives, and in snack food products [6]. The protein content in legume grains varies according to species to species [7].

A large number of legume seeds are produced in different regions of Nepal. The commonly available legumes in different regions of Nepal are chickpeas, pea, mung bean, lima bean, broad bean, lentil, soybean, and common bean. Hence, these legume species are taken as the study materials in this research. But, peoples are not aware of consuming these legumes as a sustainable and inexpensive meat alternative for vegetarian and nonvegetarian people. These legumes are not well incorporated into diets, especially in developing countries like Nepal. The purpose of this research is to study the legume seeds' composition and quantify the amount of phenolic and flavonoid content and antioxidant and antidiabetic activity of eight legume species grown in the different regions of Nepal. To the best of our knowledge, this is the first attempt to study the seed composition analysis, antioxidant activity, antidiabetic activity, and estimation of total phenolic and flavonoid content in the eight species of legume seeds growing in different regions of Nepal.

## 2. Materials and Methods

### 2.1. Chemicals and Equipment

Most of the chemicals used in this research were of analytical reagent grade. Methanol (Fisher Scientific), acetone (Fischer Scientific), and dimethyl sulphoxide (DMSO) (Merck) were purchased from Kathmandu Nepal. Folin-Ciocalteu's phenol reagent (FCR), *α*-amylase enzyme, and acarbose (Sigma-Aldrich, St. Louis) were also purchased from Kathmandu Nepal. Chemicals and reagents like gallic acid, quercetin, ascorbic acid, 1,1-diphenyl-2-picrylhydrazyl (DPPH), NaNO_2_, AlCl_3_, KOH, and NaOH were of analytical grade (Sigma Aldrich, St. Louis) and purchased from Kathmandu Nepal. Electric grinder, mortar and pestle, Kjeldahl digestion instrument (Hanon SH420F), silica crucible, distillation flask (Borosil), Muffle furnace (400-1200°C Box Furnace, Michigan), Soxhlet extraction apparatus (Sigma-Aldrich Z556203), digital weighing balance (GT 210), hot air oven (Griffin-Grundy), rotatory evaporator (Buchi RE 111) with a water bath (Buchi 461), and UV-visible spectrophotometer (WPA, supplied by Philip Harris Shenstone, England) were used to perform the laboratory works.

### 2.2. Collection of Legume Grains

The commonly used well-known eight species of grain legumes were collected from the different districts of Nepal in June by consulting the local farmers. The list of samples used in this study is presented in Table 1.

**Figure 1:**
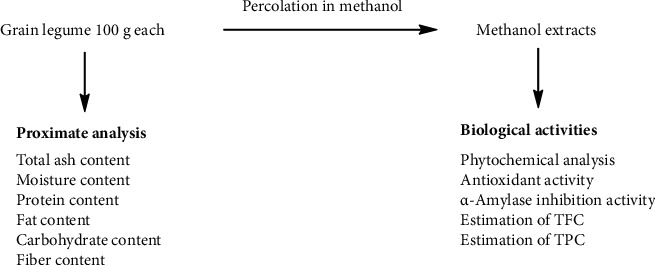
Flowsheet diagram of the composition analysis and biological activities of legume grains.

The collected legumes were washed with tap water to remove the contaminants. The legumes were grounded into powder form in an electric grinder and stored in clean plastic bags. The grounded legumes (100 g each) were kept separately in clean and dry conical flasks. 300 mL methanol was added to each flask and kept for three days with frequent shaking. The mixtures were decanted and filtered with the help of a cotton plug and thus obtained filtrates were concentrated in a rotary evaporator at a temperature below 55°C. The filtrates were kept in a beaker wrapping with aluminum foil to allow the solvent to evaporate completely. The semisolid/solid methanolic extracts were stored at 4°C until required for the biological activities and proximate composition analysis. The flowsheet diagram of the study is shown in Figure 1.

### 2.3. Phytochemical Analysis

The methanolic extract of legume grains was qualitatively analyzed to know the presence or absence of secondary metabolites such as alkaloids, polyphenols, flavonoids, saponins, and tannins by the color differentiation methods [8, 9].

#### 2.3.1. Analysis of Alkaloids

The grain legume extract is treated with the picric acid solution, an orange-red precipitate indicating the presence of alkaloids.

#### 2.3.2. Analysis of Flavonoids

The solution of grain legume extract was warmed, and the metal magnesium was added. To this reaction mixture, 5-6 drops of concentrated hydrochloric acid are added and observed for the development of red color indicates the presence of flavonoids.

#### 2.3.3. Analysis of Polyphenols

The methanolic extract of legume grains is mixed with water, and to this solution, 1% (*w*/*v*) ferric chloride solution is added; the appearance of green color indicates the presence of polyphenols.

#### 2.3.4. Analysis of Tannins

The methanolic extract of legume grain is boiled in water, and to the filtrate, few drops of ferric chloride solution are added; appearance of brownish-green or blue-black coloration indicates the presence of tannins.

#### 2.3.5. Analysis of Saponins

To the methanolic extract of legume grain, 10 mL of distilled water is added and the solution is shaken vigorously; the appearance of stable persistent froth which indicates the presence of saponins.

### 2.4. Seed Composition Analysis

#### 2.4.1. Determination of Moisture

Moisture content was determined by drying the legumes flour after 24 h at 105°C in an air oven until a constant weight was obtained according to the procedure adopted by Gajula et al. [10] with a slight modification.

#### 2.4.2. Determination of Total Ash

Five grams of dry sample was taken in a tared crucible, and the content was charred for two hours at 100 ± 5°C in a hot air oven. Then, it was incinerated in a Muffle furnace at 600 ± 20°C till the content became white. The total ash content was determined by following the standard protocol described by Gharibzahedi et al. [11].

#### 2.4.3. Determination of Crude Fat

Crude fat content was determined by using a Soxhlet apparatus according to the procedure described by Gharibzahedi et al. [11] with a slight modification.

#### 2.4.4. Determination of Protein

The protein content was calculated from the nitrogen content using a nitrogen conversion factor of % *N* × 6.25 analyzed by the Kjeldahl method [11]. The 0.5 g of accurately weighed sample was transferred to Kjeldahl flask and 2.5 g of digestion mixture and 10 mL of conc. Sulphuric acid was added to it. The flask was then adjusted to the hot plate, in the exhaust tube and heated first at low temperature until the frothing ceased then heated to boil the acid vigorously and digested for 3 hours.

(1) $$\begin{array}{c} \%\text{Total nitrogen by weight}\left(X\right)=\frac{1.4\times \left(V-V_{1}\right)\times N\times 10}{W}, \end{array}$$

where *V* is the volume of standard acid used to neutralize the distillate, *V*1 is the volume of standard acid used to neutralize the blank, *N* is the normality of standard acid, *W* is the weight in g of the sample taken for digestion 1.4 is the factor, and *X* is the total nitrogen percent.

(2) $$\begin{array}{c} \%\text{Ammonical nitrogen by weight}\left(Y\right)=\frac{1.4\times \left(V-V_{1}\right)\times N}{W}, \end{array}$$

where *V* is the volume of standard H_2_SO_4_ used to neutralize the filtrate received with the sample, *V*1 is the volume of standard H_2_SO_4_ used to neutralize the blank, *N* is the normality of the standard acid, and *W* is the weight in g of the sample taken. Thus, the crude protein can be calculated by the formula,

(3) $$\begin{array}{c} \text{Crude Protein},\text{Percent by weight}=6.25\times \left(X–Y\right), \end{array}$$

where 6.25 is the protein factor used, *X* is the total nitrogen percent, and *Y* is the total ammonical nitrogen percent.

#### 2.4.5. Determination of Crude Fiber

The crude fiber was determined according to the procedure described by Gajula et al. [10] with some modifications.

#### 2.4.6. Estimation of Carbohydrate

Total carbohydrates were calculated by difference: 100–(%moisture + %crude protein + %crude fat + %ash content), according to the procedure described by Gharibzahedi et al. [11] with some modification.

### 2.5. Evaluation of DPPH Radical Scavenging Activity

The DPPH radical scavenging activity of legumes extracts was evaluated according to the procedure described by Khan et al. [12] with a slight modification. The working solutions were prepared by serial dilution in 50% DMSO, and the DPPH solution was added to each solution. The mixture was then shaken well and incubated in dark for 30 minutes at room temperature. The absorbance was recorded at 517 nm where the control was measured using the same procedure except for the legume extracts. The degree of discoloration of the DPPH solution indicates the scavenging potential of the legume extracts. Each experiment was done in triplicate, and the DPPH RSA was calculated by using the following equation:

(4) $$\begin{array}{c} \%\text{scavenging}=\frac{A_{b}-A_{t}}{A_{b}}\times 100, \end{array}$$

where *A*_*b*_ and *A*_*t*_ are the absorbance of the blank and test sample solutions, respectively. The concentration corresponding to 50% inhibition (IC_50_) was calculated graphically by plotting the percent radical scavenging against the different concentrations of the solution.

### 2.6. Antidiabetic Activity

The *α*-amylase inhibition activity of legume extracts was evaluated according to the procedure described by Kusano et al. [13] in which the undigested starch due to enzyme inhibition was detected at 630 nm (blue, starch-iodine complex) spectrophotometrically.

(5) $$\begin{array}{c} \%\text{Inhibition}=1-\frac{\left(A_{1}-A_{2}\right)}{\left(A_{4-A3}\right)}\times 100\%, \end{array}$$

where *A*_1_ is the absorbance of the incubated mixture containing a sample, starch, and *α*-amylase, *A*_2_ is the absorbance of an incubated mixture of sample and starch, *A*_3_ is the absorbance of the incubated mixture of starch and amylase, and *A*_4_ is the absorbance of the incubated solution containing starch only. The inhibitory concentration (IC_50_) was calculated graphically by plotting the concentration against the percent inhibition.

### 2.7. Determination of Total Phenolic and Flavonoid Content

The total phenolic content in eight legume varieties was measured by Folin-Ciocalteu's phenol colorimetric method based on the oxidation-reduction reaction according to the procedure described by Gajula et al. [10]. Briefly, a calibration curve was constructed by taking gallic acid as standard (*R*^2^ = 0.99). The solution of legumes extracts was mixed with the FCR reagent and the sodium carbonate solution. The content was incubated in dark for 15 minutes, and the absorbance was recorded in triplicate. The total phenolic content was calculated by a calibration curve of gallic acid and expressed as mg·GAE/g of legume flour. The total phenolic content was calculated as

(6) $$\begin{array}{c} C=\frac{cV}{m}, \end{array}$$

where *C* is the total phenolic content, (mg·GAE/g); *c* is the concentration of gallic acid established from the calibration curve (mg/mL), *V* is the volume of the legume extract (mL), and *m* is the mass of the legume extract (g).

The total flavonoid content in eight legume species was estimated by aluminum chloride colorimetric assay according to the procedure described by Gajula et al. [10]. The total flavonoid content was calculated by a calibration curve (*R*^2^ = 0.99) of quercetin as standard, and the values are expressed in mg·QE/g of legume extract.

## 3. Results and Discussion

Phytochemical analysis showed the eight legumes species grown in different regions of Nepal are found a good source of secondary metabolites such as flavonoids, polyphenols, alkaloids, saponins, and tannins.

### 3.1. Seed Composition Analysis

The results of the proximate composition analysis of eight legume species are shown in Table 2.

The present study showed that Nepalese common legumes are rich sources of protein among crops. Protein content among these eight species of legume was found to range from 20.63 ± 0.02% in broad bean to 39.09 ± 0.03% in soybean, which shows that the protein content in soybean was significantly high among these eight legume species. Moisture content was found a bit low as compared to other food, the range of moisture was from 10.37 ± 0.01% in soybean to 14.05 ± 0.05% in lima bean. The fat content in these legumes was found considerably low. The lowest fat content was found 0.86 ± 0.02% in pea whereas soybean has a significantly high-fat content 24.73 ± 0.15%. The crude fiber ranges from 3.52 ± 0.04% in common bean to 9.65 ± 0.03% in chickpeas. Chickpeas was found to be more fibrous than the other seven legumes. Ash (minerals after burning) content ranges from 2.71 ± 0.02% in common bean to 3.75 ± 0.02% in broad bean. Legumes are rich sources of carbohydrates, and the highest percentage of carbohydrates was found 55.36 ± 0.04% in lima bean to 16.86 ± 0.03% in soybean. Soybean can be preferred to consume by people whose blood sugar level is high. The results of the legume seeds composition analysis are consistent with the previously reported results in the common legumes.

There is a challenge for researchers to find out if there are typical genetic-ecological implications of particular storage compounds in the grain legume species. Our results provide an overall indication that soybean seed composition is distinctly different from other legume species, in having much higher protein and oil content and much lower carbohydrates. One of the most important questions is how to trade off the balance between biosynthesis of proteins and carbohydrates in the seeds in a species-dependent manner. The higher nutritional composition of soybean seeds can be implemented due to the more complex network involved in the biosynthesis of storage proteins than carbohydrates [14].

From the results of the proximate composition analysis of eight species of legumes, soybean is a rich source of protein 39.09 ± 0.03% and fat 24.73 ± 0.15% as compared to the previously reported results that were 37.69% protein and 28.20% fat [15]. The proximate composition analysis of these eight common legumes grown in Nepal showed the protein content in a similar range to the previously reported results but varied among the legumes types and the different geography.

The protein content in these eight legumes was found almost the same as the protein content in chickpea (17.57 ± 0.97%), yellow soybean (37.29 ± 1.99%), and kidney bean (42.22 ± 2.35%) as reported by Shun-Cheng et al. [16].

The total ash content and the fat percentage reported in *Vicia faba* by Millara et al. [17] were 3.77% and 3.40%, respectively, which was found comparable to the results of this study. The carbohydrate and moisture content in *Phaseolus lunatus* was found significantly high 55.36 ± 0.04% and 14.05 ± 0.05%, respectively. The results of the present study were found a little higher as compared to 54.31-59.64% and 9.19-11.83% reported by Yellavilla et al. [18]. The crude fiber 9.65 ± 0.03% found in *Cicer arietinum* is significantly low as compared to the previously reported results 12.2% by Wallace et al. [19]. This variation in the proximate composition in grain legumes is due to the altitude variation and climatic conditions, rainfall, soil condition, exposure to sunlight, research method, and also due to the time length of the experiment as compared to the previously reported results in the same species as reference [16].

### 3.2. Antioxidant Activity

**Figure 2:**
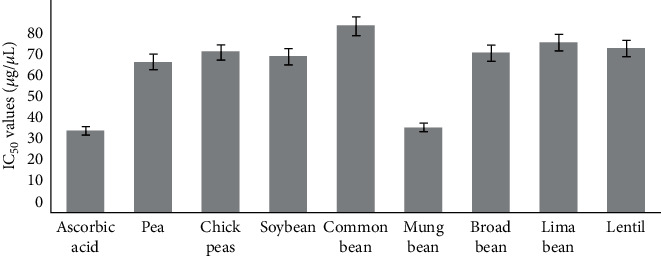
The IC_50_ values for antioxidant activity of legumes grains and standard ascorbic acid.

The antioxidant activity of methanol extract of eight legume varieties was evaluated by DPPH radical scavenging assay taking ascorbic acid as standard. Inhibitory concentration IC50 values were calculated graphically by plotting the percentage inhibition against the concentrations. The results of antioxidant activity are shown in Figure 2.

Antioxidant activity is inversely proportional to the IC50 values, i.e., extracts or fractions of compounds having low IC50 values are potent antioxidants. The IC50 value of pea was found to be 56.33 ± 0.13 *μ*g/mL, chickpeas 59.88 ± 0.26 *μ*g/mL, broad bean 59.60 ± 0.24 *μ*g/mL, common bean 69.74 ± 0.08 *μ*g/mL, soybean 58.33 ± 0.26 *μ*g/mL, mung bean 31.60 ± 0.06 *μ*g/mL, lima bean 63.66 ± 0.89 *μ*g/mL, and lentil 61.50 ± 0.16 *μ*g/mL. Since these values are comparable with the IC50 value of standard; ascorbic acid 30.30 ± 0.06 *μ*g/mL. The results showed the legumes grown in the different regions of Nepal are the potent natural antioxidant playing a significant role to minimize the oxidative damage caused by free radicals generated in the human cells.

The DPPH radical scavenging activity shown by the legumes in this study was found higher than that of the previously reported results. The antioxidant activity showed by soaked beans was reported in the range of 11.6 to 94.2% [20]. The radical scavenging activity of legumes and split pulses are in a range between 42.9 and 57.1 mg TE/100 g; cowpea showed significantly higher antioxidant activity while lower was observed for black gram dal [21].

The IC50 value of methanol extract of mung bean 31.60 ± 0.06 *μ*g/mL was found lowest among all the legumes which are very near to that of ascorbic acid 30.30 ± 0.061 *μ*g/mL, whereas common bean 69.74 ± 0.89 *μ*g/mL was found to be highest. It means mung bean has the strongest antioxidant activity whereas common bean has the lowest antioxidant activity among the eight legume varieties grown in Nepal. Various polyphenolic compounds have been reported to exhibit antioxidant activities because of the reactivity of the phenolic moiety, scavenging free radicals via electron donation, or hydrogen donation [22].

### 3.3. Antidiabetic Activity

**Figure 3:**
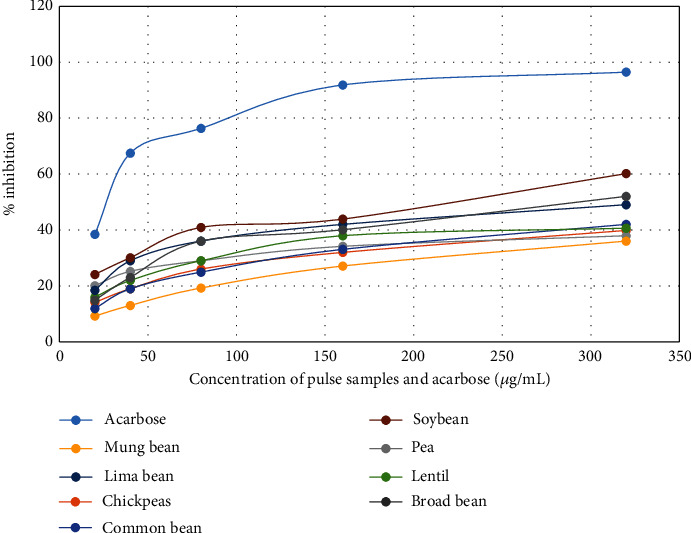
The *α*-amylase inhibitory activity against the concentrations of legume grains and standard acarbose.

The results of *α*-amylase enzyme inhibition activity were calculated as the inhibitory concentration IC50 graphically by plotting the percentage inhibition against the concentration of legumes using acarbose as the standard. The plotting of percentage inhibition against the concentrations of pulse samples is shown in Figure 3. The inhibitory concentration IC50 against *α*-amylase enzyme inhibition activity was found to be 401.46 *μ*g/mL shown by pea, 376.38 *μ*g/mL by chickpea*s*, 425.75 *μ*g/mL by mung bean, 351.83 *μ*g/mL by common bean, 349.73 *μ*g/mL by lentil, 275.32 *μ*g/mL by lima bean, 217.38 *μ*g/mL by soybean, and 264.69 *μ*g/mL by broad bean. The *α*-amylase enzyme inhibition concentration of legume samples was found much higher as compared to the standard acarbose with IC50 52.76 *μ*g/mL. The results showed that legumes are mild sources of natural antidiabetic agents.

The alpha-amylase enzyme inhibition activity showed by the legume seeds was found lower as compared to the previously reported results. The inhibitory concentration IC50 value of soybean 217.38 *μ*g/mL is lower as reported by Ademiluyia and Oboha [23] 526.32 ± 10.2 *μ*g/mL, which showed the Nepalese legume soybean is the source of potent natural *α*-amylase inhibitory agent. This variation in enzyme inhibition activity is due to the climatic condition and altitude variation, types of the species, and environmental conditions such as rainfall, soil condition, exposure to sunlight, and experimental methods.

### 3.4. Total Phenolic and Flavonoid Content

The total phenolic and flavonoid content in the pulse samples are shown in Table 3.

The results showed that legumes are rich sources of phenolic and flavonoid compounds. The soybean is rich in phenolic content 46.65 ± 1.00 mg·GAE/g and lima bean 30.64 ± 1.50 mg·GAE/g is a poor source of phenolic content. The results showed flavonoid content in eight studied common legume grains lies in the range of 135.35 ± 10.88 in common bean to 191.70 ± 8.73 mg·QE/g in soybean. Phenolic compounds including flavonoids are the most widely distributed secondary metabolites related to defense response in plants and contribute as a source of dietary antioxidants to promote human health [24]. It has been reported that the total phenolic content equivalent to gallic acid of different legumes is in the range of 38.6 to 542.7 mg/100 g [25]. Sreeramulu et al. [25] reported that the phenolic content was in the range of 62 to 418 mg·GAE/100 g. The value was found highest in black gram dal while green gram dal had the least. In green gram dal (without husk), it is found to be 55.2 mg·GAE/100 g TPC, whereas Sreeramulu et al. [25] detected 62.4 mg·GAE/100 g. One of the previous studies reported that the high antioxidant activity of pulses with seed coat was due to large amounts of phenolic and flavonoid compounds located in this part, and it can be used as the source of natural antioxidants [26].

Previously, it has been reported that flavonoid content in pulses and split pulses ranged from 18.3 to 344.7 mg RE/100 g. Cowpea (red, small) showed a significantly higher flavonoid content as observed in TPC whereas the lowest value was observed in field bean (white) [23]. It has been reported that flavones, flavonols, and proanthocyanidins from methanolic extracts of the seed coat and cotyledon of lentils and dark peas contributed the most antioxidant capacity to the seed coat [26].

## 4. Conclusions

The current study revealed the proximate composition analysis, antioxidant activity, antidiabetic activity, and determination of total phenolic and flavonoid content by using different methods in the eight legume species grown in different regions of Nepal. The results indicated that phenolic and flavonoids are the major contributors to the antioxidant properties of legumes. Legume seeds are good sources of natural antioxidants in which mung bean exhibited high antioxidant activity as compared to the eight legume species. The study revealed legumes exhibit mild antidiabetic activity whereas soybean has high *α*-amylase enzyme inhibition as compared to the eight legumes species. Legume seeds are rich in phenolic and flavonoid content in which soybean has the highest phenolic and flavonoid content. The proximate analysis showed that among the eight legume species studied, broad bean has high ash content whereas common bean has the least. Crude fat is found maximum in soybean and minimum in chickpeas. The protein content is found maximum in soybean and minimum in broad bean. Chickpeas have high crude fiber but common bean has the least. Carbohydrate is found high in lima bean whereas the soybean is the least source of carbohydrate. The present study revealed the tested legume seeds are a potential source of valuable nutrients. Soybean as a whole is distinctly superior to the other grain legumes tested based on seed phenols and flavonoids that result in better health-promoting properties. The study also revealed that soybeans growing in Nepal could be used as a sustainable and inexpensive meat alternative for vegetarian and nonvegetarian people.

## Figures and Tables

**Table 1:** The list of legume samples with scientific names, common names, and collected regions.

Legume grains	Local name	English name	Sample collected district
*Cicer arietinum*	Chana	Chickpeas	Dang
*Pisum sativum*	Kerau	Pea	Banke
*Vigna mungo*	Kalo mash	Mung bean	Dhading
*Phaseolus lunatus*	Ghyu Simi	Lima bean	Solukhumbu
*Vicia faba*	Bakulla	Broad bean	Sindhupalchowk
*Lens culinaris*	Masuro	Lentil	Banke
*Glycine max*	Bhatmas	Soybean	Kavreplanchowk
*Phaseolus vulgaris*	Rajma	Common bean	Makawanpur

**Table 2:** The proximate composition analysis of eight legume grains per 100 g (%) of samples.

Legume grains	Moisture	Total ash	Crude fat	Protein	Crude fiber	Carbohydrate
Pea	13.60 ± 0.05	2.96 ± 0.01	0.86 ± 0.02	25.03 ± 0.01	6.27 ± 0.15	51.26 ± 0.02
Chickpeas	10.63 ± 0.01	3.22 ± 0.01	1.24 ± 0.01	21.70 ± 0.02	9.65 ± 0.03	53.31 ± 0.02
Mung bean	10.63 ± 0.01	3.2 ± 0.02	2.07 ± 0.01	25.45 ± 0.03	5.27 ± 0.01	53.54 ± 0.02
Common bean	13.61 ± 0.05	2.71 ± 0.02	0.93 ± 0.01	25.57 ± 0.01	3.52 ± 0.04	53.71 ± 0.02
Soybean	10.37 ± 0.01	3.12 ± 0.01	24.73 ± 0.15	39.09 ± 0.03	6.08 ± 0.04	16.86 ± 0.03
Broad bean	12.25 ± 0.05	3.75 ± 0.02	1.94 ± 0.02	20.63 ± 0.02	7.12 ± 0.02	54.40 ± 0.03
Lima bean	14.05 ± 0.05	3.12 ± 0.01	1.26 ± 0.02	22.11 ± 0.03	4.27 ± 0.03	55.36 ± 0.04
Lentil	11.99 ± 0.04	3.23 ± 0.01	2.41 ± 0.02	24.81 ± 0.03	6.37 ± 0.45	51.55 ± 0.02

**Table 3:** Total phenolic and flavonoid content in eight different legume grains.

Legume samples	Total phenolic content (mg·GAE/g)	Total flavonoid content (mg·QE/g)
Pea (*Pisum sativum)*	33.14 ± 0.50	137.12 ± 4.3
Chickpeas *(Cicer arietinum)*	43.65 ± 0.50	177.64 ± 4.55
Mung bean *(Vigna mungo)*	45.32 ± 1.25	173.16 ± 6.72
Common bean *(Phaseolus vulgaris)*	39.31 ± 1.26	135.35 ± 10.88
Soybean (*Glycine max)*	46.65 ± 1.00	191.70 ± 8.73
Broad bean (*Vicia faba)*	32.31 ± 1.03	149.10 ± 4.43
Lima bean *(Phaseolus lunatus)*	30.64 ± 1.50	145.03 ± 8.12
Lentil *(Lens culinaris)*	41.65 ± 1.32	160.24 ± 4.07

## Data Availability

The data used to support the finding of this research are included in the article.
